# Smoking Cessation Experience in Indonesia: Does the Non-smoking Wife Play a Role?

**DOI:** 10.3389/fpsyg.2021.618182

**Published:** 2021-07-14

**Authors:** Dyah A. Ayuningtyas, Marrit A. Tuinman, Yayi S. Prabandari, Mariët Hagedoorn

**Affiliations:** ^1^Department of Health Psychology, University of Groningen, University Medical Center Groningen, Groningen, Netherlands; ^2^Department of Health Behavior, Environment Health and Social Medicine, Faculty of Medicine, Public Health and Nursing, Universitas Gadjah Mada, Yogyakarta, Indonesia; ^3^Center for Health Behavior and Promotion, Faculty of Medicine, Public Health and Nursing, Universitas Gadjah Mada, Yogyakarta, Indonesia

**Keywords:** smoking, smoking cessation, spouses, qualitative research, Indonesia, Asian – other

## Abstract

**Objective:**

More than 63% of Indonesian men are smokers, and smoking has long been a part of Indonesian culture and the concept of masculinity in Indonesian culture. Given the pro-smoking environment, we were interested in examining why smokers would willingly quit and whether their wives played a role in their quitting process as social factors are mentioned second most frequent as a reason for quitting smoking.

**Design:**

In-depth interviews.

**Method:**

We interviewed 11 couples (*N* = 22)—ex-smoking husbands and non-smoking wives—in Yogyakarta, Indonesia. The data were analysed by using the thematic analysis approach.

**Results:**

Four themes were discussed: (1) reasons for stopping smoking, (2) the process of quitting smoking, (3) the wives’ attitudes toward smoking, and (4) the families’ attempts to make the (ex-)smokers quit. The most commonly cited reasons for quitting were family and personal motivation. The (ex-)smokers preferred to quit without assistance and in private. The wives’ attitude toward smoking ranged from dislike to tolerance, and most did not know when their husbands were trying to quit. Both husbands and wives reported that the wives did not influence the smoking cessation process.

**Conclusion:**

Indonesian ex-smokers often had multiple reasons for quitting smoking. The process was typically difficult and kept private. While wives had little influence on the cessation process, they provided support and could institute a smoking ban in the house.

## Introduction

At 63%, the prevalence of smokers among men in Indonesia is the highest in the world (World Health Organization, 2019c). In contrast, only 5% of the women smoke, making smoking almost exclusively the behaviour of men in Indonesia (World Health Organization, 2019c). With a population of over 266 million (World Health Organization, 2019c), the number of current smokers is estimated at over 70 million (World Health Organization, 2015). The Indonesian government has been making efforts to reduce this number such as introducing mandatory warnings on cigarette packaging, partial bans on advertisements and implementing smoke-free areas in some regions (Amalia et al., 2019). Despite these efforts, the prevalence of smoking has not declined in the last decade (Amalia et al., 2019). Moreover, smoking has been a part of Indonesian cultural practice from the early to mid-1900s (Barraclough, 1999; Nichter et al., 2009b), with the result that smoking is accepted as a standard or even an essential part of being a man (Ng et al., 2007).

Additionally, smoking has many associated benefits among men, such that it helps to reduce stress, improve concentration and control emotion (Nichter et al., 2009b). Tobacco advertisements also utilise these associated benefits, selling cigarettes as aids to achieve self-control and inner strength, along with other cultural ideals prescribed for men (Nichter et al., 2009b). Smoking is such a substantial part of daily lives that cigarettes and tobacco are the second-largest consumption expenditure (12%) in Indonesian households after ready-made food and beverages (Badan Pusat Statistik, 2020). In such a pro-smoking environment, it is interesting to examine why some smokers would willingly quit in the first place. A previous study found that health is the most common reason for quitting smoking (McCaul et al., 2006). However, it does not move all smokers, considering some smokers continue to smoke despite ill health (Jonsdottir and Jonsdottir, 2007; Nichter et al., 2016). Studying healthy ex-smokers’ motivation for quitting could therefore be useful in developing strategies to reduce smoking rates.

Social factor is the second most common reason to quit smoking (McCaul et al., 2006). In the context of smoking, social factor is a peculiar one. On one hand, smoking is a highly social behaviour and a valuable tool or an essential part of social interaction in many Asian countries (Nichter et al., 2009a; Mao et al., 2013), including Indonesia. It is common for men to offer or distribute cigarettes during social gatherings (Nichter et al., 2009b), and they might fear harming social relationships if they refrain from smoking (Ng et al., 2007) or enforce a no-smoking rule for visitors in their houses (Nichter et al., 2010).

On the other hand, the influence of significant others and changes in social norms are among the forms of social factor that could impede smoking. It is common for married men, especially fathers, to quit smoking out of a desire to set an example for their children (Im et al., 2015), or with their children’s health in mind (Mao et al., 2015). They also experience a stigma for being smoking fathers (Greaves et al., 2010).

Additionally, in household settings, the space in which smoking fathers’ are allowed to smoke is often restricted due to considerations of morality and the desire to protect their child (Oliffe et al., 2010), or a request from their wives (Mao and Robinson, 2016). Spouses, specifically non-smoking ones, have been found to have a positive influence on smoking cessation. Smokers with a non-smoking spouse have a more pronounced intention and greater odds of quitting smoking than smokers with smoking spouses (Rüge et al., 2008; Jackson et al., 2015). Spouses also often try to promote healthy behaviours such as quitting smoking (Umberson, 1992). This act is an example of social control—an attempt to influence another’s behaviour (Lewis and Butterfield, 2007). Previous studies in Europe and North America have shown that social control is associated with positive health behaviours, including reducing or quitting smoking (Ochsner et al., 2014; Craddock et al., 2015).

However, spousal influence on smoking has not received much attention in Asian countries, although the ratio of male to female smokers in Asia can be as high as 28:1 (Sinha et al., 2016), which suggests that many smokers have non-smoking wives. A few studies in India and Indonesia suggest that wives’ impact on husbands’ smoking is limited (Nichter et al., 2009a, 2010). A previous survey in Jogjakarta, Indonesia, had found that 70% of the women disliked their husbands’ smoking habit in the house, but their requests for their husbands to smoke outside were often ignored (Nichter et al., 2010). It is important to note that, in many Asian countries, it may be socially less acceptable for wives to try to force their husbands to quit smoking as this could appear disrespectful (Nichter et al., 2009a). The request might anger the husbands or even result in violence against the wives (Padmawati et al., 2018).

Nevertheless, some studies conducted in China have shown how women managed to overcome the patriarchal barriers to exert their influence on smoking management at home, using their role as the health guardian of the family to this end (Mao, 2013, 2015; Mao and Robinson, 2016). They managed to restrict home smoking prior, during and after pregnancy (Mao and Robinson, 2016), and joined forces with their mothers-in-law to gain more power at home (Mao, 2015). Similarly, in the case of the Indonesian women, those who felt powerless against their husbands’ smoking habit were on board with an initiative to create a community-wide ban and extensive campaigns against smoking inside the house that were sponsored by Quit Tobacco International and the Ministry of Health (Nichter et al., 2010). The women supported this ban as the request not to smoke inside, then became a social norm that was supported by the community instead of a personal one (Nichter et al., 2010; Padmawati et al., 2018). As Indonesians endorse a collectivist culture, following what others do is highly essential, and smokers reported feeling embarrassed if they were caught smoking inside after the ban (Padmawati et al., 2018). The women also felt more empowered to request their husbands to smoke outside the house (Padmawati et al., 2018). These findings suggest that there are ways within the cultural constraints that women could employ to influence their husbands’ smoking. Therefore, we are interested in examining whether and how Indonesian wives try to influence their husbands’ smoking cessation and whether it is effective.

We are also interested in the process of quitting smoking itself. Quitting smoking is notoriously difficult, with smokers who want to quit often having to try multiple times (McCaul et al., 2006; Caponnetto et al., 2013). Previous studies have reported increased success in quitting among smokers who receive smoking cessation assistance (Cahill et al., 2013). Despite the positive evidence, the majority of smokers still choose to quit unassisted (Edwards et al., 2014; Smith et al., 2015b), especially Asian smokers (Mao and Bottorff, 2017). In Indonesia, the government provides a free quit line for counselling (Direktorat Pencegahan dan Pengendalian Penyakit Tidak Menular, (nd).) but does not cover nicotine replacement therapy (NRT) or other cessation services (World Health Organization, 2017). It is not known how many smokers utilise the service provided, and, to our knowledge, there is currently no data on how Indonesian smokers choose to quit.

Therefore, this study aimed to examine the experience of Indonesian ex-smokers in quitting smoking, specifically their reasons for quitting smoking, and whether and how their wives played a role in it. The current study has an exploratory nature, and as such, we decided to conduct a qualitative study using in-depth interviews to gain an understanding of the experiences of ex-smokers’ and their wives. We did not follow a specific theoretical framework in studying the quitting smoking experience, but we did follow a social control framework to study the wives’ role.

## Materials and Methods

### Recruitment

Prior to recruitment, we obtained a research permit from the province and municipality of Yogyakarta. The study was approved by the Medical and Health Research Ethics Committee of Universitas Gadjah Mada, and registered under the number KE/FK/0123/EC/2018. Participants were recruited from various neighbourhoods in the city of Yogyakarta and Sleman Regency, Indonesia. Convenience and snowball sampling methods were used to recruit participants, who were approached via a community gatekeeper, personal contacts and online advertisement. The community gatekeeper had the data about the ex-smokers in the neighbourhood from the primary health care clinics (puskesmas), and we approached them face-to-face or via text messages to introduce the study and asked whether they were willing to participate. The participants were also asked if they could recommend anyone who would fit the inclusion criteria. In addition, the first author posted the recruitment poster on her social media accounts (Facebook, Instagram, Twitter and WhatsApp) as a public post with the request to share and forward to others. Most of the participants were recruited through the community gatekeeper (*N* = 12), eight by snowball sampling, and two through social media. None of the participants had any relationship with the first author although one of the wives was acquainted with one of the interviewers. In this case, we assigned this particular interviewer to interview the participant’s husband to ensure that no interviewer had a personal relationship with the interviewee, and we could still proceed with simultaneous interviews.

The inclusion criteria were: (1) being in a relationship consisting of an ex-smoker husband and a non-smoking wife, (2) cohabiting, (3) 18 years old or older, and (4) not suffering from smoking-related chronic illnesses such as bronchitis. The ex-smoker had to meet additional requirements, namely: (5) he was a daily smoker, (6) had quit smoking less than five years ago, and (7) had quit smoking after the start of the relationship. In addition to the 22 participants we recruited, we approached three more potential participants whom the community gatekeeper knew had quit smoking. However, we did not recruit them for the following reasons: two participants had quit smoking for more than five years and one participant did not live with his wife. We only included participants with a recent smoking cessation experience (i.e., less than five years) to reduce the likelihood of recall bias. Neither ex-smokers nor non-smoking wives with chronic illnesses that have a strong association with smoking, i.e., related to the respiratory tract, were considered for this study. Those could be highly influential to the decision to quit and overshadow one of the focuses of this study, namely the wife’s role in quitting.

### Data Collection

The data collection were conducted in April 2018 and May 2018, using in-depth interviews. The interviews were conducted by four female interviewers (i.e., the first author and three research assistants). The first author is a Ph.D. student, two of the interviewers had an anthropology background and worked in a health research centre and the last interviewer was an Educational Psychology Master’s student. All interviewers were trained in observation and interview techniques, and the first author and the interviewers from the health research centre had conducted or been involved in smoking research projects prior to this study. Previous studies advised to have male interviewers for conducting an interview with male participants to establish better rapport (Williams and Heikes, 1993; Oliffe and Mróz, 2005). However, we did not have noticeable issues connecting and interviewing the male participants.

All participants gave their consent for being interviewed and audio-recorded. The interviews ranged between 19 and 73 min, with an average of 38 min, and were conducted in a private or semiprivate area at a time chosen by the participants, mostly at the participants’ houses (18 interviews) and workplaces, which were businesses owned by the participants (four interviews). In most interviews, only the interviewer and participant were present at the location, except for the interviews at the workplaces, where customers were sometimes present. We decided to interview the ex-smokers and their wives simultaneously, in separate places, for a few reasons. First, conducting the interviews simultaneously increased the efficiency. It eased the burden and time investment of the participants, so the interviewers did not take too much of the participants’ time. Second, by conducting the interviews separately, we could keep the conversations confidential. However, there are two interviews (one couple) that had to be conducted in the same place, one after another, due to the lack of space in the participants’ houses.

As we were interested in the wives’ role and influence, we cross-checked the wives’ stories with the ex-smokers in an attempt to gain data source triangulation (Carter et al., 2014). The cross-check process was done in all interviews except three, where the wives said they had not done anything to change their husbands’ smoking (*N* = 2) or had not noticed the quit attempt (*N* = 1). We found no discrepancy between these wives’ claims and their husbands’ perception.

All interviews were conducted once, and the participants were given a small gift of a hand towel and a sticker to thank them for their participation. We used similar sticker designs as the ones used for the smoke-free home interventions (Nichter et al., 2010; Padmawati et al., 2018), namely three variations of the message “I am proud that my house is smoke-free.” We continued the recruitment process until the data saturation was reached. In this study, the data saturation was defined as no new content regarding the motivation, process and wives’ role in smoking cessation.

The interviews were semi-structured, where the topics included the ex-smokers’ previous smoking habit, smoking cessation experience, wives’ attitude toward smoking and wives’ role in smoking cessation. The interview questions were pilot tested in three couples beyond the study participants, and are available as a Supplementary Material. The pilot participants and their data were not included in the final analysis.

### Data Analysis

We transcribed all recordings and analysed the data using the thematic analysis approach, where patterns or themes in the data were identified, analysed and reported (Braun and Clarke, 2006). We interviewed ex-smokers and wives separately to obtain both sides of the experience. The data were coded in Indonesian by the first author and a second coder who had not been involved in the interview process or the research team, to achieve investigator triangulation (Carter et al., 2014).

The interviews were coded by using OpenCode 4.03, a freely available tool for coding qualitative data generated from text information, including interviews (ICT Services and System Development and Division of Epidemiology and Global Health, 2013). The first author translated four interviews (from two randomly chosen couples) and the codes from Indonesian to English. The translation facilitated a discussion with the other authors. After all authors agreed on the initial codes, the first author and second coder continued coding the data in Indonesian. The first author and the second coder coded half of the interviews independently and discussed and resolved any discrepancies. The remaining interviews were coded in a round-robin format to increase efficiency. In this format, the first author coded the interviews and the second coder reviewed and noted any differences with the initial codes from the first author, which were later discussed and resolved. After the interviews were coded, the codes were categorised, and the themes were built by using the categories. The final codes, categories and themes were translated from Indonesian into English by the first author. The participants’ quotes in this article were translated by the first author and examined by two independent bilingual Indonesian-English speakers to ensure the quality of the translation.

## Results

### Sample Characteristics

We interviewed 11 heterosexual couples (*N* = 22) for this study. The characteristics of the study participants are available in Table 1. The ex-smokers’ mean age was 49 (range: 34–61 years) and they had quit smoking on average for 3 years. The wives’ mean age was 47 (range: 30–56 years), and the couples had been together on average for 21 years. The majority of ex-smokers and wives had middle-level education. In this article, we refer to all participants using pseudonyms.

**TABLE 1:** Characteristics of the study participants.

Characteristics	Ex-smoker (*N* = 11) No. (%)	Non-smoking wife (*N* = 11) No. (%)
Education		
Low (0–6 years of formal education)	2 (18)	–
Middle (7–12 years of formal education)	5 (45)	7 (64)
High (>12 years of formal education)	4 (27)	4 (36)

**Characteristics**	**Ex-smoker (*N* = 11) Mean (SD)**	**Non-smoking wife (*N* = 11) Mean (SD)**

Age (years)	49 (10)	47 (9)
Number of cigarettes per day	22 (10)	–
Relationship duration (years)	21 (11)	21 (11)

We discussed four themes in the interviews, namely the motivation to quit smoking, the process of quitting smoking, the wives’ attitude toward smoking and lastly, the family’s attempts to make the (ex-)smokers quit. The majority of ex-smokers said they stopped smoking out of their own volition, and that they had kept their smoking separate from their family. It means that the ex-smokers smoked outside the house and avoided being around their children and wives if their families were also outside. Their family also refused to partake in their smoking activity such as helping to buy cigarettes. Hence, according to the ex-smokers, the wives seemed to only play small roles in the smoking cessation process. The following themes further describe the reasons for smoking cessation, the process, and whether and how the wives and the rest of the family played a role in it.

### Motivation to Stop Smoking

Ex-smokers mentioned various motivators in their decision to stop smoking. The family was among the most frequently cited, specifically health concerns for the (grand)children, or not wanting their (grand)children to smoke in the future.

*Then after my grandchild was born and my son [said], ‘Please don’t smoke when holding the baby’, ‘Okay’. My son, then my daughter-in-law [said] ‘Please don’t smoke for a bit [when holding the baby]’, like that. Why were there so many restrictions, [I] just wanted to hold my grandchild, so I threw [the cigarettes] away. I still had six cigarettes then, [I told my friend] ‘Take these [cigarettes]’, I had a friend passing by.*… *I wanted to hold my grandchild, ‘Surely I don’t love cigarettes more [than my grandchild]’, I thought. ‘I have to stop! For my grandchild.’*… *It was the intention [that helped me persist in quitting], my intention to quit was for my family’s health especially [my] children [and] grandchildren, for me, [my] family, and my descendants.* (Sudin, ex-smoker)

*I didn’t want to be seen smoking in front of my kids it’s something that I [make sure of] from the beginning, because I had my own history of how I learned to smoke [from my family] so no I don’t smoke in the hope that my children also won’t smoke.*… *So that in the future I can forbid them [to smoke]. I don’t smoke, and I mean a smoking father is like, I mean I used to[,] when my parents forbid me to smoke when I first started smoking, [I said to them] ‘But Dad smokes [, why can’t I?].’* (Hari, ex-smoker)

Here, Sudin and Hari had different reasons to quit smoking although they both talked about their families. Hari was acting on behalf of his task as a father, who is supposed to be a good role model for his children. On the other hand, although Sudin concluded that his motivation to quit was for his family’s health, his turning point was when he was cornered by the rules imposed by his son and daughter-in-law, which forced him to revaluate his relationship with smoking.

Many of the ex-smokers mentioned personal or religious motivations as their consideration for stopping smoking. For example, some stopped smoking because they felt smoking was hindering their active hobbies, or they wanted to be a better person. Religion was mentioned, with some participants believing that smoking is *haram* (forbidden) in Islam. Another participant said that since he had become a *hajji* (someone who had performed the pilgrimage to Mecca, considered a respectable title in Indonesia), he had to be held to a higher standard than the ordinary people, which included being a non-smoker.

A few ex-smokers also mentioned social motivation such as stopping because they felt challenged by a friend’s or colleague’s successful smoking cessation.

*I had some fellow veterinarian friends who did not smoke. So I was like ‘why can they not smoke?’ so that [made] me motivated to um, start quitting.*…*I was motivated why, how come they could [not smoke]. I should be able to [as well], right. So I tried to [quit smoking]*. (Wawan, ex-smoker)

However, as most men in Indonesia smoke, it is not uncommon for ex-smokers to have smoking friends who tease them for quitting or try to lure them back to smoking. A few ex-smokers had relapsed because of their smoking friends or colleagues. The ex-smokers did not seem to take their friends’ sabotage personally, but instead described it as harmless, friendly banter.

Plenty of smokers also mentioned financial motivation such as quitting smoking due to a change of job, financial situation or the ever-increasing price of cigarettes.

*[Smoking] costs a lot of money. Right, the price of smoking keeps increasing. It used to be only*… *A few*… *A few thousand now it’s eighteen thousand a pack. Lately [before I quit smoking I] smoked two packs [a day]. For example a da [I] bought [cigarettes] in the morning, in the afternoon there would be none left. [I] bought [cigarettes] again, and so on. If added up how much is that, thirty*… *More right? (Interviewer: Thirty six.) Right. Every day. For twenty years.*… *(laughs) How many motorcycles could I get from that money? (laughs)* (Tariq, ex-smoker)

Although multiple ex-smokers acknowledged that they spent a lot of money smoking, plenty of them also commented that they did not save money from quitting smoking or justified the money they spent on cigarettes. Despite lamenting that he could have bought a house with the money he had spent on cigarettes, one ex-smoker said that smoking was a basic need for him at the time. The other ex-smokers concurred, and additionally, smoking was also considered an essential requirement for working. Smoking when working helped them to concentrate or stay awake when working late. Money spent on cigarettes was as important as food expenses, or a necessary cost for productivity’s sake, and therefore was not a waste. Secondly, many smokers tried to quit smoking by snacking, which sometimes might cost them more than cigarettes.

Health was also a common motivation for ex-smokers to stop smoking. Several smokers mentioned minor health problems such as coughing or feeling not too well in the morning. An ex-smoker also mentioned fear caused by seeing ill smokers as one of his motivations to quit. Another participant suffered from hypertension and acknowledged its role in motivating him to quit smoking, but it was not his primary reason. However, it is essential to note that due to the inclusion criteria for this study, none of the participants had any illnesses that have a strong association with smoking in Indonesia, i.e., respiratory-related illnesses.

While almost all ex-smokers mentioned more than one motivator and consideration to quit smoking, it was difficult to determine what the most common important reason was. For example, despite their family being mentioned as influential in their decision to quit smoking by many ex-smokers, only two had stopped smoking solely for that reason. A few ex-smokers had also stopped smoking more than once, ranging from two to four attempts until they successfully quit, and the reason behind every cessation attempt could differ. Additionally, although some ex-smokers had been confronted with reasons to stop smoking well before they had finally quit, it seems that the reasons need to come at the right time before the ex-smokers finally embraced them and commenced on their cessation. As an ex-smoker described:

*I chose [to quit] a month before [my second child’s birth]. I really started*… *The intention to quit was really strong, a month before [the birth] and then that was it, I quit. I mean I just quit! Completely [I] didn’t smoke at all. At all. (Interviewer: What was the difference with when you were having your first child?) I was thinking. If we have a young child, if we smoke, if we want to hold the child we have to wash hands, brush teeth, and I yeah that. I was also thinking about the risk factors for my children. So I had the intention [to quit]. (Interviewer: With the first child, you had not thought of those*… *risk factors?) Not yet. (laughs) Maybe [I had] but my ego was still too big. Maybe it was also because of my age. (laughs) The older I got I became more*… *You know.* (Rian, ex-smoker)

It is also important to note that eight couples, i.e., 16 participants, interviewed in this study lived in the neighbourhoods that implemented the smoke-free household initiative (Nichter et al., 2010; Padmawati et al., 2018). However, when asked whether they were familiar with the initiative, only six participants (four ex-smokers and two wives) were familiar with it. Among the six, one wife mentioned becoming more aware of the dangers of smoking, and her husband tried to quit smoking for a week after the smoke-free home declaration. One ex-smoker and his wife said it supported him to keep his smoking outside the house. One ex-smoker said he did not care for the initiative, and the last one was opposed to it.

### The Process of Quitting Smoking

The majority of the ex-smokers reported facing difficulties in quitting smoking. Various complaints and barriers, such as joint pain, weight gain, feeling irritable, lethargic and a craving for cigarettes, were voiced in the interviews. Many advocated quitting cold turkey as the key, going as far as saying that there is no point in cutting back. However, several ex-smokers reported that cold turkey had not worked for them. These ex-smokers chose to reduce the number of cigarettes they smoked in a day or switched to cigarettes with a lower nicotine content until they could quit smoking altogether. No one tried formal interventions, such as NRT, except for one who tried to self-medicate using apple vinegar as an alternative treatment.

*I read an article that apple vinegar is good for quitting smoking, while I was in Malang* (a city in Indonesia known for its apples) *I thought maybe this was destiny that I was directed here to quit smoking (laughs).*… *My friend gave the apple vinegar to me umm then I made my own therapy I juiced apples, Malang apples and for me it was quite effective.*… *I stopped for three months.* (Hari, ex-smoker)

All ex-smokers reported switching to snacking or keeping themselves busy to distract themselves from the temptation to smoke. As previously mentioned, some of the ex-smokers had relapsed more than once. The ones who had relapsed mentioned their unsupportive friends or work environment as the reason for their failure to sustain smoking cessation.

When talking about the essential factors that helped them to quit smoking, all ex-smokers stressed the importance of intention. Some mentioned they had tried to quit smoking before without entirely wanting to, which had not worked. Although they admitted that the smoking cessation process was still difficult, their intention kept them moving forward.

Despite the difficulties, the ex-smokers kept their smoking and cessation process to themselves. They did not let their wives or friends know when they were trying to stop smoking. A few even kept quiet until weeks after they had successfully managed to stop. When asked to elaborate, the ex-smokers did not have a specific reason for this.

*[Interviewer: So you pledged to stop once you have a son. Did you tell your wife about it?] No*… *I just stopped. It just happened out of nowhere. I mean I stopped, well I just stopped, that’s it.* (Rian, ex-smoker)

When the wives were interviewed, some mentioned they had noticed their husbands’ attempt to quit smoking but also chose not to question or comment on it. Similar to the ex-smokers, they did not have any particular reason for doing so except for one who said she did not want to draw special attention to the attempt to avoid her husband feeling self-conscious. She further elaborated:

*I noticed “he stopped smoking? He hasn’t been smoking” but I kept quiet, so I, I pretended not to notice at the beginning, so I am the type that pretends not to know because if I asked [him] “Did you quit?” he would start smoking again so I pretended not to, not to pay attention. (Interviewer: Why did you think if you commented “oh you’ve quit smoking” your husband would start smoking again?) Well with that, I, I maybe I compared it to myself, like if I have a bad habit and get told off I like to get on (the bad habit) again because I don’t like it when [I get told off]*… *I mean if I’m not doing the bad habit anymore don’t pay too much attention to it just treat it as a normal thing, for example [if I exercise] then [husband] goes “You went swimming woop woop!” then I get annoyed, so I just treated him like me but turns out he actually wanted the attention.* (Dina, wife)

Dina’s experience was related to other ex-smokers’ quitting smoking process, where they did not tell anyone, including their wives, about their smoking cessation effort. Neither did they tell their wives about their expectations on support, or in Dina’s husband’s case, attention. The lack of communication led Dina to treat her husband’s smoking cessation as she would prefer to be treated, which backfired as her husband started smoking again out of his disappointment that Dina did not pay enough attention to him.

The other wife said she did not have a specific reason for not admitting that she noticed her husband’s cessation, but she had previously mentioned that she was afraid her husband would get angry if she tried to hide or throw his cigarettes away.

*I noticed [that my husband quit smoking] but I didn’t ask. “Why is he not smoking?” I wondered, then I checked [his pockets] and oh there were only very few cigarettes.*…*After two months I kept checking but there was no cigarettes anymore*… *I found two candies [instead of cigarettes]. I found two [candies] but I still didn’t ask. After a few months I asked why he no longer bought cigarettes.*…*I just wanted to check [the cigarettes]. [I] didn’t want*… *To throw it away or hide it I didn’t want to do it.*… *I mean when you want to*… *Reprove you have to do it little by little right? If [I] was insistent [husband] would get angry.* (Sumi, wife)

Sumi believed she should just be patient and wait until he would stop on his own, and she would quietly check her husband’s pocket to keep notes of how much he had smoked on the day. Her decision to quietly observe and wait might be a reflection of the patriarchal Javanese culture that places her as her husband’s subordinate (Hermawati, 2007). As previously mentioned, a wife who insisted on her husband’s smoking cessation could be considered inappropriate and challenging his authority (Padmawati et al., 2018), since she was not supposed to meddle and should obey her husband (Hermawati, 2007).

### Wives’ Attitudes Toward Smoking

*Smoking is bad but tolerable*

The wives’ attitudes ranged from tolerating to disliking their husbands’ smoking. They complained of their poor financial situation due to the smoking expenses, their worry about their husbands’ and children’s health, and how smoking affected the cleanliness of their homes.

*[Husband] could smoke a pack a day every time he sat in front of the computer [working] then the cigarette butts would be scattered and [I] had to clean up. (laughs) And [he] would move around [from] there [to] here there would be ashtrays. [I] would find cigarette butts everywhere.* (Mira, wife)

The ones who tolerated their husbands’ smoking cited their husbands’ excuse that smoking was essential for their work. We have previously mentioned that the ex-smokers reported smoking to be a necessity when working, although the participants with office jobs mentioned it less. The majority of our participants were business owners and artists, and those who were not spent the majority of their working hours outside, where there was no smoking restriction. The participants with office jobs often smoked when they had to work late at home. On top of justifying smoking as a work necessity, some ex-smokers also framed smoking as a vice that their wives simply had to accept.

*At that time my excuse [to smoke] was that I um didn’t have any other extensive hobby I mean I never did anything wrong, and it’s just smoking are you really going to not allow me this?* (Rahman, ex-smoker)

Several wives deemed this view acceptable, as they felt that their husbands’ smoking was not problematic enough to concern them. Given that their husbands did not smoke an excessive amount, a few wives decided to ignore their husbands’ smoking, as illustrated by one of the wives:

*I never paid attention to my husband[’s smoking] because I spent my time in the kitchen. So no ignorance is the best. Really. So I don’t know how much of a heavy smoker my husband was I didn’t really pay attention.*…*My husband, I don’t know when [he] bought cigarettes, I also don’t know what cigarettes [he] smoked. But my husband didn’t smoke that much.* (Indah, wife)

Another wife also mentioned not knowing much about her husband’s smoking habit as he never asked her for money. There does not seem to be a clear limit on how much their husbands had to smoke to be considered excessive, and arguably, the wives might never consider it to be so as they often underestimated how much their husbands smoked. For example, Indah estimated her husband to smoke about half a pack of cigarettes a day, whilst her husband recounted that he could smoke two packs a day.

Lastly, some wives reported being afraid that their husbands would get angry if they complained about smoking or think it was inappropriate for wives to tell their husbands what to do. They also considered the fact that smoking is a part of cultural practice in Indonesia and the notion that a man is supposed to smoke, which both wives and ex-smokers mentioned:

*(Interviewer: Your wife had never complained about your habit when you still smoked?) It’s normal isn’t it [for men] to smoke, especially my wife is also from East Java. All her uncles smoke, [they are] all heavy smokers.*…*[Wife] mostly said “Well men are supposed to smoke right that’s the right way.”* (Danang, ex-smoker)

All wives acknowledged that smoking had little to no benefits, but no wives considered smoking to be a problem in their relationship unless there was a tremendous financial concern related to it.

*We used to [fight] a lot [about smoking] because of money issues I think that’s the number one [issue] back then. Rather than buying cigarettes, I was annoyed I [said] ‘Why don’t you [use the money spent on cigarettes to] buy food for your children’ or something like that, I remember, it’s just money issues. Our business was not doing well, it’s been four years that it’s this slow, you see.* (Lina, wife)

As previously mentioned, cigarettes or other tobacco products are the second biggest consumption expenditure in Indonesian households (Badan Pusat Statistik, 2020), which shows how essential buying cigarettes is for men. One of the ex-smokers concurred:

*You can always get some money for cigarettes, [you can] find coins [around the house] to buy cigarettes, but I can’t save money. (laughs)* (Adam ex-smoker)

*Quitting Smoking Is Not a Big Deal*

When talking about their husbands’ smoking cessation, all of the wives said they were happy but did not consider it an extraordinary achievement. There was no excessive appreciation or excitement when the (ex-)smokers stopped. The wives mainly described that they were happy with the benefits such as the houses became cleaner, their expenses decreased and their husbands and family were healthier. There were two wives who mentioned that they were very grateful and admiring their husbands’ strong will to persist in quitting smoking. However, they did not seem to communicate it to their husbands:

*I was surprised [that my husband quit smoking]. Maybe I should throw a* syukuran… (Note: syukuran is a celebratory event held by Muslims that involves reciting prayers as a group, followed by a feast) *(laughs) I was confused. And grateful that he really quit. [But I] wasn’t [reacting] excessively or anything. (Interviewer: “You didn’t go (excitedly cheers) ‘aaaaaa!”’) No no, I was I was and still am, I just feel like I admire [him] more “how could he stop just like that!” That’s it. That’s it.* (Tantri, wife)

When questioned about their wives’ lack of apparent excitement, the ex-smokers explained that their wives were happy, but did not recall them being overly excited or ecstatic about it.

*(Interviewer: How did your wife react when [she] knew you quit smoking.) Nothing much maybe she kept the happiness inside. (laughs) I don’t know. But she was definitely happy. (Interviewer: Maybe there is a change from) Nah it’s just the same. She stopped nagging, that’s the difference. (laughs)* (Bimo, ex-smoker)

It is unclear why the wives, especially the ones who were ecstatic about the cessation, decided not to express it to their husbands. Considering many wives had been asking their husbands to quit smoking for years, and the fact that many ex-smokers faced difficulties in quitting smoking, we expected a bigger reaction from both parties. However, no ex-smoker considered their cessation to be something that warranted special treatment or celebration, except for one. It might be that the wives considered smoking as an annoying habit, but not a deal breaker. Thus, their husbands’ cessation was simply nice to have instead of worthy of a celebration. It is also possible that the wives were hesitant to celebrate early as some ex-smokers relapsed after quitting for some time. Meanwhile, the ex-smokers might not perceive their cessation as something special due to the Javanese cultural expectation to be humble and to keep a low profile (Sukarno, 2010). The Javanese people also mainly use indirect communication, and as such, they are expected to be able to read between the lines (Sukarno, 2010). The ability to read between the lines might explain how Bimo could confidently say his wife was happy about his cessation although, according to him, she never showed it.

### Family’s Attempts to Make the (Ex-)smokers Quit

*Wives have little influence on smoking cessation*

The ex-smokers previously mentioned that their wives did not have a role in their smoking cessation, and the majority of the wives concurred. The wives did mention trying to persuade or demand their husbands to quit smoking, throwing their husbands’ cigarettes away, or enforcing smoking rules before their husbands stopped smoking.

*When I was pregnant [I told my husband] once we have the child you should stop smoking*… *Yes, [he] said. In the beginning*… *[Child] was already born he still smoked but outside [the house], fine I guess.* (Mira, wife)

These attempts to make their husbands stop smoking often fell on deaf ears. Wives mentioned that their husbands would ignore them, or they caught their husbands smoking after saying that they had stopped smoking. Seeing no change in their husbands’ smoking, they eventually resorted to relying on their husbands’ conscience to quit. Wives also tried a more supportive approach to assisting their husbands in quitting smoking such as providing an alternative to cigarettes. This approach was indeed acknowledged and considered helpful by the ex-smokers. It is important to note that the wives’ effort was only useful once the husbands had already decided and embarked on their smoking cessation attempt. Wives and ex-smokers agreed that wives could not do much to invoke smoking cessation as they had walked into the relationship fully aware that their husbands were smokers.

*Personally I don’t really like smo um*… *It’s not healthy. But*… *[My] husband had always been if [he was] asked to quit smoking, before we met he already smoked right.*…*We argued [about smoking] but we didn’t fight [about it], no. And he, he once*… *Mm said, “You knew from the beginning that I smoked right,” like so.* (Ririn, wife)

On the other hand, the children were more inclined to speak of their disdain for smoking. Both ex-smokers and wives reported their children complaining and demanding of their fathers that they quit smoking. Some children also took matters into their own hands, for example, by hiding or throwing the cigarettes away.

*My child even, I couldn’t find [the cigarettes] until now, no idea where she threw them away, even the match [as well].*… *I woke up and suddenly the cigarettes were gone, I woke up and asked “where are my cigarettes, [daughter]?” “No idea.” My wife was also probably in on it.* (Wahyu, ex-smoker)

*Ex-smokers’ reaction to families’ attempt*

No ex-smokers directly said no to their wives’ requests, opting to stay quiet or give excuses and promises they had no intention of keeping. The Javanese society values harmony above everything (Irawanto et al., 2011), and considers directly expressing their refusal to be impolite and potentially hurtful (Sukarno, 2010). Staying quiet, giving excuses or hiding their smoking are therefore preferable than being honest.

*I promised I would quit if we had a son.*…*But [I] would say [to wife] “I already, I already quit but just sometimes I smoke”*… *I um told my wife I already smoked less but I didn’t really. She caught me [smoking] many times, then I just immediately threw [the cigarette] away. (laughs)* (Adam, ex-smoker)

Adam added that his wife gave up trying to make him quit smoking after observing his unkept promises, something that was also voiced by the other wives. Adam, like most of the ex-smokers in this study, did not tell his wife when he finally quit smoking, hence most wives were not involved in the cessation process. Some ex-smokers also downright considered their wives’ attempts to be of no help at all.

*I mean, I don’t mean to speak ill of my wife but really, I’m sure she didn’t help*… *She just reminded me [to stop smoking] but the percentage [of it helping my smoking cessation] was small.* (Sudin, ex-smoker)

On the other hand, although ex-smokers mostly ignored their wives, attempts from children were considered more acceptable. Children had more leeway in voicing their disapproval compared to wives. As previously mentioned, some children would throw away their fathers’ cigarettes or threaten them. Although this confrontational approach would be unacceptable from the wife, ex-smokers reported that their children’s attempts evoked a more emotional response. A previous study found that in Indonesian and Indian society, it might be best to leave these attempts to daughters, as sons might also appear disrespectful if they tried to do the same thing (Nichter et al., 2009a). We had only noticed a difference between daughters and sons in one ex-smoker, whose daughter threatened him that he could not play with his grandchild if he kept smoking, whilst his son tried a non-confrontational approach. The other participants did not mention any gender differences. Not all children had a strong negative reaction toward their fathers’ smoking, but health warnings or requests to quit smoking from the children were treated differently from the ones that came from the wife.

*It definitely hit right in if it’s [my] child [asking me to quit smoking]. If it was [my] wife I would just ignore it. (laughs)* (Sudin, ex-smoker)

## Discussion

This study examined the smoking cessation experience of Indonesian ex-smokers and their wives’ role in it. Our findings provide an insight into the reasons for quitting, the process, and the wives’ and family members’ attitude and attempts to make the ex-smokers quit smoking. Many of the ex-smokers cited more than one reason for quitting smoking, and although family pressure was often mentioned, all of the ex-smokers had made the decision to quit themselves. The ex-smokers vouched for different methods, with some preferring cold turkey and others choosing to reduce their cigarette consumption until they managed to stop altogether. Most of the time, the process and experience were kept private although not secret. The private nature of the exercise meant that other people, including the wives, did not get to play a great role in smoking cessation. Almost all of the wives agreed that smoking did not have any benefits, but they tolerated or ignored it for cultural reasons. When it comes to attempts at making the ex-smokers quit smoking, the children were more outspoken and had a more significant emotional impact than the wives.

The social network, such as friends and family (specifically the children and grandchildren), and religion were the most commonly cited motivation to quit smoking. Friends and family played different roles in motivating (ex-)smokers to quit smoking, with friends (inadvertently) providing competition and family providing social control. Friends, specifically ex-smoker friends, served as a challenge to the (ex-)smokers to prove they also could quit smoking. Similarly, another study among smoking men found that smokers were highly motivated by the idea of a friendly competition to quit smoking, as it would provide both a challenge and support (Bottorff et al., 2018). However, Canada has a significantly lower smoking rate compared to Indonesia at 21% (World Health Organization, 2019b), and smoking is a stigmatised behaviour in Canada (Greaves et al., 2010). Although we found the friendly competitive nature to quit smoking in some of our participants, friends could also be the downfall of a smoking cessation attempt.

Meanwhile, family ignited the protector role. Previous studies have found that fathers change their smoking behaviour in order to be a responsible father who protects his children (Greaves et al., 2010). The family could also directly pressure the ex-smoker to quit. While previous studies often reported spousal pressure to quit smoking (Bottorff et al., 2010; Kwon et al., 2015), pressure from children is less common. Nevertheless, a study in Pakistani and Bangladeshi British smokers reported similar pressure from their children (Bush et al., 2003; White et al., 2006), which led them to relocate their smoking or try to quit. While the ex-smokers in this study reacted better to the children’s intervention than the wives’ intervention, there is a concern that the intervention might not be welcomed as it could be considered culturally inappropriate (White et al., 2006; Nichter et al., 2009a). It is also considered culturally inappropriate for wives to push for their husbands’ smoking cessation, and the wives fear it would elicit their husbands’ anger (Nichter et al., 2010).

The religious motivation was mentioned by Muslim ex-smokers, citing that smoking is forbidden or frowned upon in Islam. Being religious has been associated with a lower likelihood of smoking in various studies (Kim et al., 2000; Hussain et al., 2019) or as a supporting factor in smoking cessation, including smokers coming from Muslim-majority backgrounds (Naing et al., 2004; White et al., 2006; Yong et al., 2013). However, the smoking rate in Muslim and Muslim-majority countries remains remarkably high (Ghouri et al., 2006), and it is important to note that the link between religion and smoking differs between religions (Yong et al., 2013; Hussain et al., 2019). We did not ask about other participants’ religious affiliation, thus we could not be sure whether this motivation was present specifically in Muslim ex-smokers, or that the interpretation that Islam looks down on smoking was a personal opinion.

Lastly, health was found to motivate the ex-smokers to quit. This is hardly surprising as health is the most common reason for quitting smoking (McCaul et al., 2006). However, it is interesting as, due to the inclusion criteria, none of the ex-smokers in this study had any illnesses that were strongly related to smoking. The few who quit smoking for health reasons had respiratory problems that they rated as minor, and the one who quit after being ill suffered from a non-smoking-related illness. Despite the mildness of the health complaints, it was enough to raise their awareness of the fact that quitting smoking would improve their health. The participants were aware that smoking was not healthy, especially those who lived in the smoke-free home neighbourhoods had been exposed to health education and campaigns. However, we noticed that it was not a concern until they experienced it first-hand.

With regard to the process of quitting smoking, more ex-smokers advocated quitting cold turkey than cutting back on smoking. None of the ex-smokers reported seeking or receiving professional help or medication. Quitting smoking unassisted is found to be the norm in smokers across the world (White et al., 2006; Smith et al., 2015a; Mao and Bottorff, 2017), the belief being that the application of willpower should be sufficient to allow someone to quit smoking (White et al., 2006; Fu et al., 2007; Morphett et al., 2015). A similar belief was also present among the ex-smokers in this study. Everyone stressed the importance of intention, which should generate the willpower and the strength to withstand smoking temptation.

Historically, the tobacco industry has been washing its hands of tobacco addiction, stating that smoking is a choice that one can simply leave behind should one want to White et al. (2013). From the gender perspective, willpower is linked to autonomy and decisiveness, the idea being that men who are unable to quit are weak and effeminate (White et al., 2013). Admitting that willpower alone is not enough to successfully quit smoking might be considered to go against cultural (Nichter et al., 2009b) and Islamic values (White et al., 2006; Nichter et al., 2009b), as it shows that one lacks self-control in one’s desires or cravings. For the same reason, Indonesian smokers were also more reluctant to refer to their smoking as an addiction (Nichter et al., 2009b). These factors could be behind the decision to quit without assistance despite the great difficulties this poses.

The private, solitary nature of smoking cessation reported by the ex-smokers in this study is similar to another study where smokers perceived quitting smoking as something deeply personal and individual, and not something that could be helped along by others (Smith et al., 2015a). If the ex-smokers considered their smoking was a personal issue, this could explain why they did not tell anyone when they tried to quit smoking. It might also be the reason behind the wives staying quiet when they noticed their husbands’ attempts to quit smoking. Similar to how they let their husbands smoke, they also allow their husbands to quit on their own terms.

On one hand, from the gender perspective, the wives’ behaviour could be explained as emphasised femininity, where they accept and cooperate with the principles of hegemonic masculinity (Bottorff et al., 2010). Hegemonic masculinity is a dominant, idealised version of masculinity that exists within a society (Evans et al., 2011). Smoking is a part of hegemonic masculinity in Indonesia, portrayed in the social and cultural practices of male smoking and the common notion that “real” men smoke (Ng et al., 2007; Nichter et al., 2009b). The wives’ acceptance of smoking or reluctance to contest it, and they are quietly observing when their husbands were trying to quit, supported the idea that smoking and quitting fall within the husbands’ autonomy. This finding was remarkably similar to that of a previous study where the wives reported accepting smoking as their husbands’ “little thing” or compromising in order to avoid potential conflicts in the relationship (Bottorff et al., 2010).

On the other hand, the wives’ choice to stand back could inadvertently have provided invisible support for the ex-smokers. According to Bolger et al. (2000), invisible support could take shape as a supportive act that occurs outside the recipient’s awareness, such as taking over the recipient’s task without their knowledge, or an act that the recipient acknowledges but does not perceive as support. The wives’ holding back and believing that only the husbands could decide when and how to quit smoking could be the supportive act that surpassed their husbands’ awareness. The wives’ effort to relieve withdrawal symptoms or provision of cigarette replacement had also mostly gone unnoticed, or at least, was not considered an essential factor in the smoking cessation process. A study among ex-smokers previously reported that the ex-smokers perceived those actions by their spouses that afforded them the choice to quit smoking on their own terms as the only helpful, supportive act (Kwon et al., 2015). They also reported that any type of support other than sitting or holding back was considered unhelpful and could create a hostile environment in the relationship (Kwon et al., 2015), which was also a worry mentioned by the wives in this study.

To our knowledge, our study is the first to examine the smoking cessation experience of Indonesian (ex-)smokers and their spouses’ role in it. It contributes to the growing literature of smoking cessation experiences, specifically in Asian smokers. There are currently few studies involving Asian smokers, despite the fact that Asian countries, especially in the Southeast Asia region, have an exorbitantly high number of male smokers (World Health Organization, 2019a). In this study, we interviewed both ex-smokers and their wives to obtain both parties’ perspective of the smoking cessation, which enriched and improved the validity of the data.

This study has several limitations, namely that the majority of the participants lived in an area of Yogyakarta where an intervention to ban smoking at home was conducted in 2008 (Nichter et al., 2010), which involved these researchers joining various neighbourhood activities to promote smoke-free households and educate the residents on the danger of smoking. As we have previously mentioned, not all of the participants who lived there were aware or actively involved in the intervention, but it might have inclined the ex-smokers toward smoking cessation. Many of the participants, especially those in their 50s, reported only receiving education regarding the danger of smoking later in adulthood, with some mentioning they had only received the information during the intervention. Thus, the experiences of the younger generation, who are more likely to have been exposed to public health campaigns regarding smoking in school, in quitting smoking might be considerably different from what is reported in this study.

Smoking cessation intervention is still best aimed at smokers, and it should not be the family members’ responsibility to intervene. As this study shows and previous studies have shown, autonomy is important for smokers. Therefore, relying on family members to intervene is ineffective at best and, at worst, detrimental to their relationship with the smokers. Family members can instead push smokers to adapt their smoking behaviour to reduce the danger of second-hand smoking. Pregnancy is a suitable time to do so as smokers are more likely to change their smoking behaviour when expecting a child (Oliffe et al., 2010), and we also found a similar tendency in older smokers expecting a grandchild. This finding is highly relevant in Indonesia and other Asian countries where multigenerational households are common. By educating expecting parents and grandparents, the non-smoking family members could join forces to create a smoke-free house (Mao, 2015).

## Conclusion

Indonesian ex-smokers often had more than one motive for quitting smoking, with family being the most commonly mentioned motive. The smoking cessation process was often difficult and kept private, without external influence. While wives did not and could not influence the cessation process, they provided support by letting their husbands to quit on their own terms and relieve withdrawal symptoms, which had mostly gone unnoticed by the (ex-)smokers in question. Smokers’ families have a better chance of requesting the smokers to smoke outside the house and away from the family, rather than asking them to quit. The birth of a child or a grandchild could be a suitable time for such a request.

## Data Availability Statement

Requests to access the anonymised interview transcripts should be directed to DA, d.a.ayuningtyas@umcg.nl. The raw voice recording data contain the participants’ identification, and therefore cannot be shared.

## Ethics Statement

The studies involving human participants were reviewed and approved by the Medical and Health Research Ethics Committee of Universitas Gadjah Mada, Indonesia. The participants provided their written informed consent to participate in this study.

## Author Contributions

DA contributed to the study design, data collection, data analysis, and drafting of the manuscript. MT, YP, and MH contributed to the study design, data analysis, and revised the manuscript. All authors who meet authorship criteria are listed, and all authors certify that they have participated sufficiently to take public responsibility for this manuscript.

## Conflict of Interest

The authors declare that the research was conducted in the absence of any commercial or financial relationships that could be construed as a potential conflict of interest.
