# Supplementary material for: Smoking Cessation Experience in Indonesia: Does the Non-smoking Wife Play a Role?
Source: Front Psychol. 2021 Jul 14;12:618182. doi: 10.3389/fpsyg.2021.618182 (PMC8316595; doi:10.3389/fpsyg.2021.618182)
Supplement: Supplementary file 1 [file Table_1.DOCX]

***1*** *Ex-smoker*

| **Themes** | **Topic** | **Interview questions** |
| --- | --- | --- |
| Background | Daily life | Age  Education  Occupation  Relationship duration  How many people are living in the house? (kids, parents, in-laws)  Note: ask only to one of the couple (either the ex-smoker or the spouse, doesn’t need to be both) |
| Past smoking behaviour | What the smoking behaviour was like | Could you tell me how you started smoking? (why? Male identity, masculinity, just trying to blend in?) What was your journey as a smoker like? (a change in intensity, reason to smoke, etc.) What about once you became an adult, how important was smoking for you? (did it change from the time of initiation to adulthood?)  Could you tell me what your smoking behaviour was like? (get a description on whether he was a heavy smoker or not)  What about your idea of yourself now?  What were you like when you smoked? (social, relaxed, etc? Get a description of self or smoker identity)  What about now that you have stopped smoking? |
| Quitting smoking | What the quitting smoking experience was like | Could you tell me about your smoking cessation process? (have you tried and failed before? Could you tell me about it, why did you fail?)  What was the most crucial event during that time? (why, if more than one cause, which one was the most important? How?)  What was your spouse’s role in it? (supporting, controlling?)  How about other people? (were there others who had a role, and in what way?) |
| Partner’s attitude | Partner’s attitude towards the smoking behaviour | How did your spouse feel about your smoking? What gives you that impression? (accepting/against it/others, did she show it, in what way)  Could you describe how your partner usually reacted to your smoking? (behaviour: commenting, nagging, others?, emotion: annoyed, cool?) How did you feel about it? How did it affect your smoking? |
| Spousal role | The role of spouse in the smoking behaviour | What was your smoking behaviour like after you got married? (any change from before, if so, why?)  How did your spouse try to make you quit smoking? How did you feel about it? Did it affect your smoking behaviour? (what works, what doesn’t?) Did you ever hide your smoking from your spouse? (when, why, how)  Would you have liked a different behaviour? What kind?  How did your spouse help you quit smoking? How? How did you feel about it? How did it affect your smoking behaviour?  What was the most helpful thing your spouse did when you were trying to quit smoking? Why, how?  How is your relationship with your spouse after you quit smoking? (anything changed?)  If couple has kids:  What was your smoking behaviour like when your wife was pregnant, and when your children were small? |

***2*** *Non-smoking spouse*

| **Themes** | **Topic** | **Interview questions** |
| --- | --- | --- |
| Background | Daily life | Age  Education  Occupation  Relationship duration  How many people are living in the house? (kids, parents, in-laws)  Note: ask only to one of the couple (either the ex-smoker or the spouse, doesn’t need to be both) |
| Partner’s smoking | Attitude towards partner’s smoking behaviour | What are your thoughts about smoking? How do you feel about it?  Could you describe what your partner’s smoking behaviour was like? (get a description whether he was a heavy smoker or not.) What were your thoughts about your partner’s smoking? How did you feel about it? How did you show it? How did he respond to it?  Could you describe how you usually reacted to your partner’s smoking? (behaviour, emotion) How did you feel about it? How did it affect his smoking behaviour? |
| Spousal role | The role of spouse in the partner’s smoking behaviour | Did you know about your partner’s smoking before you got married?  What was his smoking behaviour like after you got married? (any change? What kind?)  Did you ask him to quit smoking? How did you try to make him quit smoking? (in other ways other than asking, e.g. nagging, complaining, dropping hints, etc.)  How did he react? How did it affect his smoking behaviour? (hiding smoking, reducing smoking, others? What worked, what didn’t?)  Could you tell me what it was like when he was trying to quit smoking? (when, why, how?) How did you help him quit smoking? How did you feel about it? How did he react? How did it affect his smoking behaviour? (hiding smoking, reducing smoking, others? What worked, what didn’t?)  Why did you think he quit smoking? (your role, others’, or other reasons?)  How is your relationship with your spouse after he quit smoking? (anything changed?)  If couple has kids:  What was your husband’s smoking behaviour like when you were pregnant, and when your children were small? How did you react to it? |
